# Exploring the Implementation of Shared Decision-Making Involving Health Coaches for Diabetes and Hypertension Self-Management: Qualitative Study

**DOI:** 10.2196/51848

**Published:** 2024-04-04

**Authors:** Sungwon Yoon, Chao Min Tan, Jie Kie Phang, Venice Xi Liu, Wee Boon Tan, Yu Heng Kwan, Lian Leng Low

**Affiliations:** 1 Duke-NUS Medical School Singapore Singapore; 2 Centre for Population Health Research and Implementation SingHealth Regional Health System Singapore Singapore

**Keywords:** decision-making, diabetes, health coach, health coaching, healthcare professional, hypertension, patient, patient-centered care, person-centered care, qualitative research, self-management, shared decision-making

## Abstract

**Background:**

An emerging focus on person-centered care has prompted the need to understand how shared decision-making (SDM) and health coaching could support self-management of diabetes and hypertension.

**Objective:**

This study aims to explore preferences for the scope of involvement of health coaches and health care professionals (HCPs) in SDM and the factors that may influence optimal implementation of SDM from the perspectives of patients and HCPs.

**Methods:**

We conducted focus group discussions with 39 patients with diabetes and hypertension and 45 HCPs involved in their care. The main topics discussed included the roles of health coaches and HCPs in self-management, views toward health coaching and SDM, and factors that should be considered for optimal implementation of SDM that involves health coaches. All focus group discussions were audio recorded, transcribed verbatim, and analyzed using thematic analysis.

**Results:**

Participants agreed that the main responsibility of HCPs should be identifying the patient’s stage of change and medication education, while health coaches should focus on lifestyle education, monitoring, and motivational conversation. The health coach was seen to be more effective in engaging patients in lifestyle education and designing goal management plans as health coaches have more time available to spend with patients. The importance of a health coach’s personal attributes (eg, sufficient knowledge of both medical and psychosocial management of disease conditions) and credentials (eg, openness, patience, and empathy) was commonly emphasized. Participants viewed that addressing the following five elements would be necessary for the optimal implementation of SDM: (1) target population (newly diagnosed and less stable patients), (2) commitment of all stakeholders (discrepancy on targeted times and modality), (3) continuity of care (familiar faces), (4) philosophy of care (person-centered communication), and (5) faces of legitimacy (physician as the ultimate authority).

**Conclusions:**

The findings shed light on the appropriate roles of health coaches vis-à-vis HCPs in SDM as perceived by patients and HCPs. Findings from this study also contribute to the understanding of SDM on self-management strategies for patients with diabetes and hypertension and highlight potential opportunities for integrating health coaches into the routine care process.

## Introduction

Lifestyle factors, including engaging in adequate physical activity and consuming a healthy diet, are important in the management of diabetes and hypertension [1-3]. However, many patients with diabetes and hypertension do not achieve optimal lifestyle targets [4-6]. Effective interventions to help patients with diabetes and hypertension should be tailored for each patient, and this may be achieved through engaging in shared decision-making (SDM). In line with the growing emphasis on person-centered care, SDM is recognized as a way to empower patients with chronic diseases such as diabetes and hypertension. SDM involves patients and health care professionals (HCPs) collaboratively making a health care decision after discussing the treatment, management, and support packages and considering the patient’s preferences, priorities, and goals [7-9]. Although there is limited research on the effect of SDM on clinical outcomes for diabetes and hypertension [10], studies invariably suggest that SDM makes a positive difference to patients in their care. This includes better treatment adherence, increased patient coping, improved knowledge attainment, higher levels of patient satisfaction, and greater empowerment [10-12].

In addition to situations requiring treatment decisions, SDM may be useful in supporting healthy behavior change [13,14]. A feasibility study on decision tools in primary care to help initiate lifestyle change among patients with or at risk of coronary heart disease has shown the potential beneficial effects of paper-based tools for SDM in initiating behavior change [15]. Another feasibility study of an internet-based decision aid to encourage lifestyle change and adherence among people at moderate or high risk of coronary heart disease was found to increase participants’ ability to make clear decisions about making changes [16,17]. However, it was suggested that further impact may have been achieved if more comprehensive implementation strategies had been available for the interventions [16,17].

Despite the evidence supporting SDM, it is not widely practiced in clinical settings due to several reasons, such as low patient self-efficacy, a power imbalance between patients and physicians, and HCP’s limited time and knowledge [12,18-20]. To overcome these communication and resource barriers, several studies proposed the inclusion of health coaches to facilitate the SDM process in chronic care through continuous counseling dialogue with patients and exploration of patients’ situations and preferences in order to make informed decisions together on treatment and lifestyle [21,22]. Health coaches are individuals who aid patients in gaining the knowledge and confidence necessary to become engaged in their care and promote communication and collaborative decisions between patients and HCPs [23]. The practice of health coaching can differ in the type of coach, their training, and their level of involvement [24]. Nonetheless, randomized controlled trials have shown that health coaching can lead to enhanced self-management of diabetes and hypertension [25,26]. Furthermore, the experiences of patients and HCPs were found to be largely positive [27-29].

Although literature has documented the effectiveness and feasibility of SDM and health coaching, the evidence primarily comes from patients and HCPs who were willing to take part in or complete interventions [30-32]. There are fewer studies that have examined the viewpoints of potential end users’ perspectives regarding their preferences for and expectations of SDM [33,34], as well as the role and relationships of health coaches in patient care practice [35]. Obtaining the buy-in of patients and HCPs is crucial when developing a robust care model. To this end, we conducted a study to gather the viewpoints of patients and HCPs to gain insight on developing strategies for SDM programs that incorporate nurse-trained health coaches in primary care.

The aim of this formative study was to explore the perspectives and preferences of HCPs and patients with diabetes and hypertension concerning the respective professional roles of health coaches and HCPs in SDM, as well as the factors that should be considered for optimal implementation of a SDM model that involves patients, health coaches, and HCPs.

## Methods

This was a qualitative study, reported following the Consolidated Criteria for Reporting Qualitative Research (COREQ) Guidelines [36].

### Setting and Participants

This study was conducted in Singapore, a multiethnic city state where the majority of the population (80%) obtains health care from the public health care system [37]. Participants were mainly from SingHealth Cluster, which is the largest regional health care system in Singapore, offering a complete range of medical care for patients, including those with diabetes and hypertension. Eligible patient participants were those aged 40 years and older, diagnosed with diabetes and hypertension, and attending public primary care clinics. We identified eligible patients from a study cohort that investigates the clinical and cost-effectiveness of a behavioral intervention delivered through mobile health [38]. The participants were then approached through a phone call with the study aim and methods explained. On the other hand, eligible HCP participants were those responsible for managing patients with diabetes and hypertension in public primary care clinics, step-down care, and secondary or tertiary centers with at least 1 year of experience. We approached potential HCP participants through email and provided background information. Purposive sampling was adopted in terms of age (patients) and clinical experience (HCPs) to maximize diversity of perspectives.

### Data Collection

We conducted focus group discussions (FGDs) with participants between February 2022 and May 2022. A semistructured topic guide was developed and subsequently pilot-tested to facilitate discussions on the roles of health coaches and HCPs in self-management, views toward health coaching and SDM, and factors that should be considered for optimal implementation of SDM that involves health coaches. All FGDs were carried out through web-based videoconferencing by facilitators (CMT and WBT) who were trained in social sciences and qualitative research and did not have a personal relationship with the participants. Each FGD session lasted approximately 90 minutes for patient participants and 60 minutes for HCP participants. No repeat interviews were conducted, and transcripts were not returned to participants for further input. Data collection and analysis were an iterative process that continued until no new themes emerged. Field notes were taken to support the contextual interpretation of the data.

### Data Analysis

All FGDs were audio-recorded and transcribed verbatim. Transcripts were checked for accuracy and thematically analyzed. A total of 2 coders (CMT and WBT) were assigned to code the patient FGDs, while 2 other coders (JKP and VXL) were assigned to code the HCP FGDs. The team adopted the 6 steps to thematic analysis suggested by Braun and Clarke [39], in which the coders first familiarize themselves with the data and generate initial codes independently before collating the codes into potential themes together. The themes were constantly reviewed, refined, and reclassified to ensure the best fit of themes to the data. Discrepancies between coders were resolved through consensus meetings involving all study team members. The FGDs were conducted until thematic saturation occurred at the 17th and 18th FGDs with patients and HCPs, respectively. Storing and managing data during data analysis was done using NVivo (version 12; QSR International).

### Ethical Considerations

This study was approved by the SingHealth Centralized Institutional Review Board (2019/2468). Participants provided verbal informed consent before the study began. The study team maintained data confidentiality by redacting personally identifiable information from interview transcripts and generating unique study identifiers, which were linked to participant identifiable information only through a password-protected file. Participants were reimbursed SG $60 (US $44.70) to defray the cost of their participation in this research.

## Results

### Participant Characteristics

Out of 89 patients approached, 39 were recruited for the study, with the most frequent reasons for the decline being difficulties in participating in web-based interviews or schedule unavailability. The recruited patients participated in 17 FGDs. Their age ranged from 43 to 68 years, with 74% (29/39) being male candidates and 64% (25/39) being Chinese. Concurrently, we approached 52 HCPs and recruited 45 HCPs who participated in 18 FGDs, with schedule unavailability as the main reason for the decline. Approximately 65% (29/45) were clinicians, followed by 16% (7/45) being nurses. The range of clinical experience in managing chronic diseases of the HCPs was from 1 year to 28 years (Table 1).

**Table 1:** Characteristics of focus group participants (N=84).

Characteristics					Patients (n=39)		HCPs^a^ (n=45)
FGDs^b^, n					17		18
**Gender, n (%)**							
		Male		29 (74)		11 (24)	
		Female		10 (26)		34 (76)	
Age (years), range					43-68		25-56
Years of clinical experience, range					N/A^c^		1-28
**Ethnicity, n (%)**							
	Chinese			25 (64)		36 (80)	
	Indian			5 (13)		4 (9)	
	Malay			9 (23)		5 (11)	
**Profession, n (%)**							
	Clinicians			N/A		29 (65)	
	Pharmacists			N/A		6 (13)	
	Dieticians			N/A		2 (4)	
	Nurses			N/A		7 (16)	
	Administrator			N/A		1 (2)	
**Diagnosis, n (%)**							
			Diabetes only		16 (41)		N/A
			Diabetes and hypertension		23 (59)		N/A

^a^HCP: health care professional.

^b^FGD: focus group discussion.

^c^N/A: not applicable.

**Figure 1:**
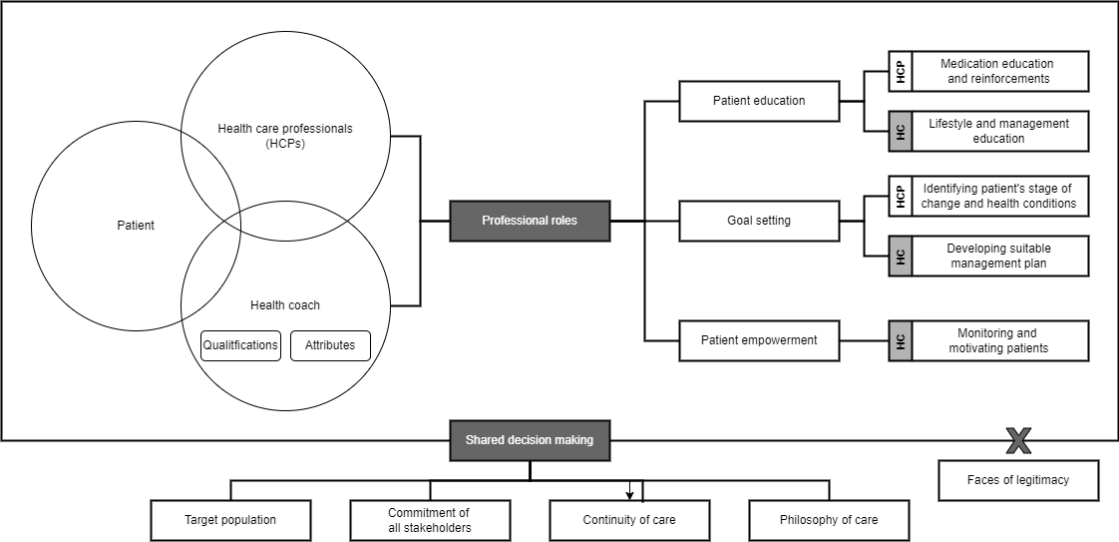
Visual summary of shared decision-making involving health coaches for diabetes and hypertension self-management. HC: health coach; HCP: health care professional.

Through our analysis, we identified three themes that represent (1) the participants’ perspectives concerning the professional roles of health coaches and HCPs in SDM, (2) the perceived importance of health coaches’ credentials and attributes, and (3) a total of 5 essential elements to be considered for optimal implementation of SDM. Figure 1 presents a visual summary that suggests how SDM involving health coaches could be applied in clinical settings to facilitate diabetes and hypertension self-management, based on the findings.

### Perceived Preference for Professional Roles of HCP and Health Coach in SDM

While there were some commonalities in the roles of HCPs and health coaches in the SDM model of care, a distinction in the extent of their responsibilities was evident.

#### Patient Education

Many of the patient participants and HCP participants recognized the shared responsibilities of HCPs and health coaches in improving patients’ understanding of their conditions in order to decide on the self-management strategies that fit best according to their individual situations and capacities. However, their expectations of the specific roles that HCPs and health coaches would play in patient education differed. The health coach was seen to be more effective in engaging patients in lifestyle education and informing patients on healthy lifestyle choices, while the HCPs are expected to educate patients on medication and alternative treatment options for their conditions. The participants explained that the difference in expectations was based on the perceived amount of time availability the professionals have with the patients and the background of the professionals.

[The health coach] is a single point of contact that I can refer to, who is an expert in this area, and I can leverage on that to achieve the goals that I want. Knowing that there is somebody associated with you, and you can engage with it helps a lot. Whether it’s about physical activity, food intake, [or] the discipline you need to get in order to achieve the goals, if I know I can reach out to someone to talk about it, it will definitely make a difference.

Patient 37, Patient FGD #13

I will tell her what the best option as a physician is, based on our guidelines. However, I will tell her other possible options if let’s say she doesn’t want the recommended option. As a physician, it’s our duty to tell patients the options they have and the pros and cons of each option.

HCP 36, HCP FGD #12

#### Goal Setting

Both patient participants and HCP participants agreed that setting actionable goals would be crucial to improving clinical outcomes. However, the brief consultation sessions in primary care settings were inadequate for patients to develop personalized care plans with their HCPs. Thus, patient participants saw the value of involving the health coach to work with them to set actionable goals while taking into consideration their personal circumstances in order to devise an appropriate self-management plan that aligns with the expectations of the HCPs. At the same time, HCPs suggested that the health coach plays an important role in identifying any lapses and bridging the gaps between the treatment offered by HCPs and the health care preferences and goals of the patients.

A doctor’s goals may be different from a patient’s goals. Sometimes it’s hard for us to assess the ideas, values, and preferences during our short 10 to 15 minutes [consultations]. If the health coach informs us, that will be good so that the patient, doctor, and health coach can be on the same page to help the patient achieve his or her goals.

HCP 36, HCP FGD #12

[A health coach should] connect with the patient to discover what the problems are and also be aware of the patient’s environment. Then align these with the expectations that HCPs might have of the patient. So, the health coach’s duty is basically to identify all of these, in order to make things easier.

Patient 25, Patient FGD #10

#### Patient Empowerment

According to patients, “feeling empowered” entailed remaining motivated to engage in behaviors that promote their health. To this end, they preferred to work with the health coaches and mutually establish achievable goals related to behaviors such as diet and exercise. The partnership that patients form with their health coach allows patients to feel supported throughout their self-management journey, which motivates them to be more engaged and adherent to the chosen care plan. Likewise, HCP participants recognized the significance of such a partnership, in which the patients are free to express their expectations and wishes for care while deciding on a care plan that would be tailored to their individual needs and preferences.

It would be more of a motivating aspect because the health coach can help set specific Agendas, target them, and communicate with patients…In this way, we are more motivated to try hard to meet the targets. Because when seeing the doctor, you know, he/she will just tell you to lose weight but [the] health coach can motivate you for sure.

Patient 25, Patient FGD #10

In an ideal setting, [health coaches] need to understand what their targets are, and what the patient thinks [of] their health conditions. From there, see what the patient is willing and able to do and what their plans are going forward.

HCP 09, HCP FGD #1

#### Importance of Health Coach’s Credentials and Attributes

Both patient participants and HCP participants emphasized the importance of health coaches’ credentials and attributes that would influence their acceptance of health coaching as part of routine patient care. Most participants mentioned that a desirable health coach would need to possess sufficient knowledge of both medical and psychosocial management of diabetes and hypertension so that health coaches could offer appropriate guidance to patients.

Health coach should be medically trained to give correct advice. I mean, apart from medication and disease management [which is the ambit of HCPs], the health coach should be able to provide psychosocial counseling.

HCP 38, HCP FGD #8

A health coach’s personal attributes were equally stressed; most participants noted that a health coach should demonstrate positive personality traits such as openness, patience, and empathy to effectively improve a patient’s willingness to consider the recommended health practices as suggested by the health coach.

How do I become open to the health coach? Firstly, I think the health coaches themselves must be very caring and full of empathy, to disarm all the unhappiness of the patient, maybe then patients will be willing to tell the coach about their story.

Patient 51, Patient FGD #17

### 5 Essential Elements for Optimal Implementation of SDM

A total of 5 elements have been identified that participants believe should be considered for optimal implementation of the SDM model of care involving health coaches.

#### Target Population

Most participants (patients and HCPs) shared that SDM should target a certain segment of patients to maximize its benefits. They believed that individuals with newly diagnosed chronic conditions and those with poorly controlled diabetes and hypertension would benefit more from the proposed SDM model of care involving health coaches. This is because these patient groups may have inadequate knowledge or support to effectively manage their conditions. Thus, the education, guidance, and discussion provided by the health coach could prepare them with the necessary knowledge to begin their self-management journey effectively.

Maybe this will help for those who just are diagnosed with diabetes, and you know, those at a loss or don’t know what to do. But if you’re working with a “seasoned” patient, they know what to do, what to expect and all.

Patient 17, Patient FGD #3

#### Commitment of all Stakeholders

Participants asserted that in order to effectively facilitate SDM and achieve the mutually established goals of improving self-management, all stakeholders involved in this communication process should be equally committed. They expressed commitment to engaging in open dialogues and establishing partnerships among all 3 parties, including patients, HCPs, and health coaches, at targeted times and through preferred modalities. While patient participants expressed a preference for frequent check-ins with the health coach (eg, monthly), HCP participants felt that meetings between health coaches and HCPs should be arranged on a case-by-case basis, depending on the urgency and complexity of the patient’s conditions due to their high workload. Most participants were open to the varied modes of communication, including in-person and web-based means to facilitate the SDM to cater to different situational needs (eg, web-based call for a brief check-in and an in-person call for an in-depth dialogue).

I think doctors need to focus on the very complex cases for communication with health coaches. We can’t put too much effort into handling every single chronic patient because the workload will be too high.

HCP 02, HCP FGD #2

#### Continuity of Care

Care continuity was identified by participants as a critical factor in facilitating SDM. Both HCP participants and patient participants expressed a preference for having the same health coach for follow-up appointments to foster a sense of rapport, continuity of care, and motivate patients to carry out the plan of care. Additionally, patient participants felt that following up with the same health coach would help them build trust with their health coach and disclose their concerns, strengths, and limitations in their self-management journey.

In a polyclinic setting, the doctor will change every appointment. If the same health coach can provide consistent support and counseling to the patient, and if the patient has someone who is checking on him, he will want to take better care of his chronic conditions.

HCP 02, HCP FGD #2

I think the health coach should be someone that at least I know and have some level of good relationship…so I can treat the coach like a friend and open up.

Patient 32, Patient FGD #9

#### Philosophy of Care

Participants highlighted the importance of open communication and person-centered inquiry to facilitate mutual understanding among all parties involved in SDM. They valued strategies that could allow patients to set personal goals, negotiate, and discuss challenges. Thus, the philosophy of care was focused on supporting patients to make informed choices and engaging patients in discussion to develop a care plan that is tailored to individuals, as opposed to simply offering generic health advice that may not be as effective in motivating patients.

Ask the patient what they want first, because if it’s not something that they want, it’s not likely that they will cooperate with us [HCPs or health coaches] even though it is what we want from them.

HCP 49, HCP FGD #18

#### Faces of Legitimacy

Notably, our interviews revealed that patients generally prioritized the advice given by their physicians because they perceived their physician’s advice to be more important and reliable than that of health coaches and other HCPs (eg, dieticians, nurses, and physiotherapists). This could pose a challenge to SDM when consensus cannot be built about the preferred self-management plan among all parties involved and patients are less receptive to exploring other treatment options and recommendations unless they are endorsed by a physician.

I’ll still take the final instructions from the doctor. Frankly speaking, the health coach might be knowledgeable in terms of some medical information, but they are still not reliable.

Patient 17, Patient FGD #3

Sometimes it also depends on which healthcare provider is approaching the patient. A lot of times, our patients defer to what we intended so if the doctors don’t say like “Oh you need to do this” then they won’t really cooperate because doctor’s recommendations take precedence over whatever other professionals are seeking to help.

HCP 49, HCP FGD #18

To mitigate this, some of our HCP participants, who are physicians, suggested that reinforcement and endorsing the advice from the health coaches and other HCPs during follow-up appointments would be important in increasing patients’ trust in other providers and promoting effective communication in SDM.

One thing that physicians like me could do is to reinforce what the health coach has taught the patients. Then the patient would realize that, Oh yes, that [advice given by health coach] is very important.

HCP 49, HCP FGD #18

## Discussion

### Principle Findings

This study explored the preferences and perspectives of both patients and HCPs on how SDM involving health coaches could help patients make informed decisions about their health and improve self-management of their diabetes and hypertension. While some perspectives varied across patients and HCPs, we identified three unified themes, including (1) the perceived preference for and expectation of the roles of HCPs and health coaches in SDM, (2) the importance of health coaches’ credentials and attributes, and (3) the 5 elements necessary for effective implementation of SDM. The findings gained from this study offered key insights to support efforts to optimally implement SDM involving health coaches for patients with chronic conditions.

The lack of patient education [40,41] and psychosocial support [42-45] can hinder patients’ ability to self-manage their diabetes and hypertension, which eventually results in suboptimal control and negative health outcomes. In this study, patient participants and HCP participants agreed that the primary responsibility of a health coach is to educate patients on healthy lifestyle choices and provide several self-management options before setting actionable goals that align with the patient’s needs and preferences, while an HCP is expected to provide medication education and offer alternative treatment options for their conditions. In this regard, SDM provides a platform for patients, health coaches, and HCPs to engage in conversations that enable information to be shared and address each party’s expectations for care [13]. In addition, the involvement of health coaches in SDM has been shown to improve self-management by fostering greater patient involvement in their care and designing care plans that take into account their unique treatment goals and preferences [46]. When patients have a better understanding of their options and have the autonomy to express their preferences and wishes for care, they are more likely to be satisfied with the eventual plan of care and adhere to it [47]. Moreover, our findings showed that the involvement of a health coach would offer patients a sense of support through their self-management journey and motivate them to take charge of their diabetes and hypertension self-care. This finding reflects previous studies that found integrative health coaching improved patients’ psychosocial outcomes, resulting in reduced perceived barriers to self-management, enhanced perceptions of social support, and ultimately improved clinical outcomes in patients with diabetes and hypertension [48-50]. The results of this study offer valuable insights into the distinct responsibilities of health coaches and HCPs in SDM and chronic disease management and highlight potential areas of emphasis in patient coaching, workflow, and collaborative efforts.

A common theme running through the FGDs was participants’ keen interest in the credentials and characteristics of health coaches. Participants stressed the importance of a health coach’s positive attitude and knowledge in both the medical and psychosocial aspects of disease management in order to engage in a partner relationship in SDM. Indeed, a health coach’s professional expertise and personal traits, such as openness and empathy, can improve the therapeutic relationship and ultimately enhance self-management skills [30,51]. Therefore, it is essential for health coaches to receive appropriate clinical training and education as well as possess a strong capacity for empathy [52]. Beyond the credentials and characteristics of health coaches, studies on SDM also stressed the importance of a supportive and caring environment with adequate interaction time as key aspects of the patient-provider partnership in chronic care [53,54]. Many of the physicians interviewed in this study often mentioned that they are unable to cover all aspects of the patient’s self-care due to the brief consultation sessions they have with the patients. The time required for information sharing and clarifying patients’ values, needs, and preferences could impact the already-pressured clinical setting [13]. Therefore, it was suggested that the involvement of health coaches in SDM would help to prioritize discussions about specific aspects of diabetes and hypertension self-management (ie, medical and lifestyle) and allow patients to benefit from the enhanced support from their health coaches, who have more time to work with them on modifying their lifestyle and achieving better control of their conditions. Our findings underscore the significance of health coaches’ competencies to ensure that health coaches can fulfill their core responsibilities and the potential benefit of involving health coaches in SDM to support patients further in their self-management journey.

Lastly, our participants believed that newly diagnosed or less stable patients could benefit the most from SDM involving health coaches and emphasized the importance of continuity of care through the same coach. They also recognized that open communication and person-centered inquiry would be crucial for improving the quality of SDM. Indeed, previous studies demonstrated that open communication and consistent coaching improved decision quality, knowledge, and risk perception among patients with diabetes and hypertension [10,55]. Despite these findings supporting the use of SDM to support healthy behavior change among patients with chronic disease, patients in this study still held a strong belief in the traditional approach of “doctor knows best” (faces of legitimacy), with many patients relying disproportionately on physicians for decisions [56]. Patients’ reliance on physicians for decisions may pose a challenge to the SDM process involving health coaches since patients may prioritize the advice of their physicians over that of health coaches. For health care institutions that wish to implement SDM for chronic disease management, we suggest distinguishing the roles of HCPs and health coaches in chronic disease management to ensure successful implementation of SDM. Institutions can also consider educating patients about the unique and valuable contributions that health coaches can make in their care [57] to reinforce their trust in health coaches. As observed in this study, the health coaches’ involvement in SDM was important in offering personalized support to patients to modify their lifestyle and self-management in order to achieve behavioral change and gain better control of their diabetes and hypertension. Future research should aim to identify factors that affect patients’ engagement and trust with health coaches to enable successful implementation of this SDM model for chronic disease management.

### Limitations

This study has a few limitations. The perspectives of health coaches were not included, which may limit the comprehensiveness of the results. Furthermore, participants’ preferences and expectations were not examined by subgroups such as HCP’s professional roles or patients’ confidence levels in self-management and cultural backgrounds. Further research focusing on these aspects may prove useful for a richer understanding of the SDM implementation. Despite these limitations, this study provided valuable insights into the SDM model of care, highlighting how patients, HCPs, and health coaches can collaborate and the factors needed to be considered for robust implementation of the SDM for patients with diabetes and hypertension.

### Conclusions

Our findings examined the viewpoints of potential end users’ perspectives regarding their preferences for and expectations of SDM from patients and HCPs. Our analysis identified the appropriate roles of health coaches vis-à-vis HCPs in SDM and underscored the importance of a health coach’s credentials and personal attributes. At the same time, the five elements for optimal implementation of SDM can be used to guide future efforts to contextualize SDM and integrate health coaches into routine primary care to support diabetes and hypertension treatment.

## Appendix

Multimedia Appendix 1 Consolidated criteria for reporting qualitative research (COREQ): 32-item checklist.

## Abbreviations

COREQ: Consolidated Criteria for Reporting Qualitative Research
FGD: focus group discussion
HCP: health care professional
SDM: shared decision-making

## Data Availability

The data sets generated during and analyzed during this study are available from the corresponding author on reasonable request.
